# F124. SEX DIFFERENCES IN OUTCOME IN FIRST EPISODE PSYCHOSIS PATIENTS: A 10-YEAR FOLLOW-UP STUDY

**DOI:** 10.1093/schbul/sby017.655

**Published:** 2018-04-01

**Authors:** Rosa Ayesa-Arriola, Esther Setién-Suero, Diana Tordesillas-Gutierrez, Benedicto Crespo-Facorro

**Affiliations:** 1 Marqués de Valdecilla University Hospital, IDIVAL, School of Medicine, University of Cantabria; 2 Centro de Investigación Biomedica en Red de Salud Mental (CIBERSAM); 3 University Hospital Marques de Valdecilla -IDIVAL; University of Cantabria; CIBERSAM; 4 Marqués de Valdecilla University Hospital, IDIVAL, Instituto de Investigación Valdecilla

## Abstract

**Background:**

Specialized early intervention programs are efficient in treating patients with a first episode of psychosis (FEP) at least after 2 years. However, few studies have examined long-term outcomes, and particularly prognostic implications of the sex of FEP patients.

**Methods:**

We aimed to investigate long-term neuropsychological and functional outcomes in female and male 10 years after the first presentation of a non-affective psychotic episode. One hundred sixty-five FEP patients, 73 women and 92 men were assessed for sociodemographic, clinical and neuropsychological information.

**Results:**

Differences in outcome between female and male based on baseline, 1-year, 3-year and 10-year follow-up information were substantial, showing women better outcomes on several variables. Schizophrenia diagnosis was significantly more frequent in men (82% vs. 62%; p = 0.01). Women were more likely than men married (45% vs. 24%; p = 0.01) and having children (41% vs. 13%); p < 0.001). Significant differences arose for social function (F= 5.469; p = 0.022) and processing speed (F = 12.66; p < 0.001). There was also some weak evidence (albeit not quite statistically significant at p < 0.05) for negative symptoms and global neurocognitive function.

**Discussion:**

Women who suffered a first episode of psychosis have better functional and neurocognitive outcomes compared to men. This differential outcome profile is important for clinicians to consider sex specific therapeutic approaches.

